# Supplementary material for: Not by the light of the moon: Investigating circadian rhythms and environmental predictors of calling in Bornean great argus
Source: PLoS One. 2021 Feb 16;16(2):e0246564. doi: 10.1371/journal.pone.0246564 (PMC7886196; doi:10.1371/journal.pone.0246564)
Supplement: Attachment — Submitted filename: PLOS Argus response to reviewers Nov 2020.docx [file pone.0246564.s002.docx]

Reviewer #1: I reviewed this paper before for Ibis and unfortunately they did not accept it - clearly the different reviewers and the editor disagreed. I thought then, and I think now, that this is a sound paper and the changes I suggested when it was under review for Ibis have been incorporated so I have nothing else to suggest.

Just fix the referencing style on page 5 (names of the authors rather than numbers).

Thank you for catching this. It is now corrected.

Vincent Nijman, Oxford, 8 October 2020

Reviewer #2: REVIEW OF MANUSCRIPT PONE-D-20-28926
This is a well-written manuscript describing the vocal activity of the Bornean great argus, a threatened species that is particularly known for its amazing visual display to attract females. The methods and analyses are adequate, and the findings are relevant for understanding more about the species’ behavior and ecology. Should be an important contribution to the field.

I have made some minor commentaries throughout the manuscript regarding some points that were unclear for me. The main issue I’d like to highlight would be to improve clarity in the statistical methods used and to consider making some adjustments that are detailed below. Finally, I’d like to commend the authors for the nice work done here.

Line-by-line commentaries
Title
When I read “ecological predictors”, I expected to find goals associated with population or interactions, as competition. But the predictors used could be names as environmental variables. Perhaps you could change to “…: environmental drivers of circadian calling activity…”.

We changed the title to the following:

Not by the light of the moon: investigating circadian rhythms and environmental predictors of calling in Bornean great argus

Introduction
L. 43-51: The opening paragraph lacks a message of what’s coming next. It highlights the prospects and research gaps of PAM, but that’s just the method. It would be more interesting to be introduced with a message related to the core ecological aspect of the manuscript. For instance, why is vocal behavior important and what are the drivers of calling activity? Then, in the next paragraphs, you could describe why we still know little about these behaviors, and how PAM can help to fill this gap.

We agree with this suggestion and we added an introductory paragraph that provides more background on the ecological question(s) we address.

L. 47: There are large-scale and temporal efforts to obtain calling behavior data, such as citizen science projects. Perhaps you could say that it requires substantial efforts, not that it is impossible.

We reworded this line as follows:

In addition, PAM allows for temporal and spatial coverage that is not generally not possible using traditional methods that rely on human observers [21].

L. 97-102: It would be interesting to see a paragraph introducing the potential effects of the lunar cycle in calling activity, in general, and also for birds.

Thank you for this suggestion. We added a paragraph that discusses nocturnal song.

Additionally, it would also be interesting if the function of these calls could be introduced. Are they courtship calls? How are they characterized? Are there differences in the function of these calls?

We added the following line (although not much is really known):

The long calls have been proposed to serve a territorial function, whereas the short calls may be used for mate attraction [49].

Methods
L. 107: Please, include the unit type of the coordinates (degrees?), indicate latitude and longitude, and datum (WGS84?).

We modified the coordinate so that they follow the correct convention for reporting in decimal degrees.

N4.964936°, E117.805116°

L. 113: Were the recordings made in stereo?

We added the following:

We recorded at a sampling rate of 16 kHz, 16-bit resolution and a gain setting of 40 dB (mono, WAV format).

L. 116: Include a few words describing how are gibbon calls (frequency range, duration, stereotyped, or not).

We added the following lines:

Detection distance for gibbon female calls which are relatively stereotyped, and have a frequency range to similar to great argus (500- 1800 Hz [59]) was shown to be ~400 m at this site using the same recording settings [60].

L. 145-146: All the monitored hours were inspected by the observer?

We added the following lines to clarify:

A single observer (DJC) identified all instances of argus calling in the LTSAs using a combination of aural and visual inspection of 24-hour periods for all hours of recordings in our dataset.

L. 147: Was the 10-day period used in the analysis?

We added the following lines:

We did not use the 10-day LTSA for detecting great argus calling events, but we include a 10-day LTSA in Figure 2 to illustrate how LTSAs can be used to investigate patterns of calling at various temporal levels.

L. 151: It is really difficult to see the differences between the two call types in the LTSA. Did you also zoomed in the spectrogram to distinguish between the call types, or used always 24-hour LTSA + aural discrimination? What are the advantages of using LTSA and “zoomed-in” spectrograms, like those available in Audacity/Raven? This information can be useful for the reader interested in using LTSA.

We appreciate the reviewer comment on this point, as LTSAs are a great tool that many researchers may not be familiar with. We added the lines to clarify.

We did our initial inspection of LTSAs over 24-hour periods as it allowed us to quickly scan through long time periods of acoustic data and identify calling bouts in the frequency range of interest (500-2000 Hz). Once we identified a calling bout in a 24-hour period, we then created short duration spectrograms to determine if the calling bout was great argus or a gibbon (as gibbon calling bouts appear similar on the 24-hour LTSA [11]); if it was a great argus call we then classified it as either a short call or a long call.

Figure 2: The resolution from the downloaded figure is not good. For instance, in figure 2.A, it is not clear what the first arrow points to, although the legend says it is a call. Particularly, if this is the resolution used to identify events of call by one observer, it raises doubts if it was possible to discriminate calls. Please, indicate in the methods if the observer used the entire range of 0-8000 Hz to screen for the calls, or the 0-1600 Hz (shown in 2.C and 2.D).

Please see response above, as we hope that it helps clarify the reviewer concern.

L. 154: Please, add full LTSA meaning.

Done.

L. 159: Add “software” after Triton.

Done.

L. 179: You could add the models and its variables in table 1 in the form of “questions” or “set” of tests. For instance,
1. 24-hour calling activity?
1.1 Presence/absence of call events ~ variables
1.2. Total number of calls ~ variables
2. Night activity?
2.1 Pres/absence of call events (18:00-06:00) ~ variables
2.2 Total number of calls (18:00-06:00) ~ variables
3. Differences in call type?
Number of calls ~call type * calling period

We took a slightly different approach to address this reviewer concern. We interpreted this comment to mean that it was difficult to follow the methods for the different analyses we did. In order to address this we added subheadings to the different sections in the methods so that they match the subheadings in the results. We hope that this makes it clearer for the reader to follow.

L. 181: Each model contains only one predictor? If so, please amend this to this sentence.

We realize that in the previous version this was not clear, so we added lines to each section describing our analyses explicitly stating which predictors we included in our models.

L. 187: Are the number of argus calls the number of notes, or call events? Please, describe this in the previous section.

We added a line in the methods to clarify:

Representative spectrograms of great argus long and short calls are provided in Figure 2C and 2D; the counts in our models indicate the number of distinct calls as shown in the spectrograms.

L. 209: Did you also included a model with intercept only (null model)? This should be done to understand how different your best model is from a null one.

Yes, and we added a line to each of the sections in the methods to clarify this.

L. 221: Also add the total number of hours/days evaluated.

Done.

L. 223: Did you measured the lengths from all calls or a subset of them?

We estimated duration for all calls.

L. 221-224: If the previous sentence is “yes”, another information that would be interesting to improve our understanding of the ecology of the great argus would be to understand if any of the meteorological variables and period of the day influence the length of calls. I know this is not included in the goal but consider doing it since you already got the data.

We have actually thought about this previously. We are currently working on a second (student-led) manuscript that is investigating fine-scale patterns of variation in call features (e.g. duration, frequency, etc.). We feel that analysis would be more appropriate for another manuscript, as it is a bit out of the scope of the current study. Especially considering we are already presenting the results of five separate analyses. In addition, it is not clear to us exactly why we would expect duration to vary with environmental variables, so we could not think of any relevant hypotheses that would justify including the extra analysis.

L. 225: You could also test if a “response” was more frequent in some period of the day or for some call type. Depending on the results, it may indicate that it can function better to male-male interactions, for instance.
We added this analysis and found that long calls elicited more of a response than short calls, and this fits nicely with their proposed territorial function.

Results
L. 250: Please, include the hours corresponding to the morning and evening periods.
L. 253: There is no information about a model representing the effect of rain in table 3.

Thank you for catching this. The information is there now.

L. 255: Here you say the model was compared with a null one, but also include that in the methods. Additionally, it is not clear if you ranked each model with a null one, or if you ranked all models + null. I think the last option is better, as we can check if there are models equally plausible.

We added a line to the methods that states:

For each set of analyses, we compared all candidate models using Akaike information criterion (AIC) adjusted for small sample sizes (AICc) using the ‘bbmle’ R package [65].

L. 256: It would be nice to see a table with the models ranked, containing delta AICc, AICc, and the weights.

We added a new Table 5 to address this comment.

L. 304: Previously, I had understood that you used presence/absence from the night period as the response variable. But why the calling period enter as a predictor here?

Thank you for catching this. We have now corrected it.

Discussion
L. 348: You could explore if there are differences in calling activity between months, and indicate, for instance, which month were the individuals engaging more in calling activity. These suggestions for new analysis help in filling the gap that is raised in this sentence (little is known about the behavioral ecology of great argus…).

We added the following line in response:

Future, long-term studies of great argus vocal behavior across years (and including during mast fruiting [69] periods) will help further our understanding of how environmental and ecological factors influence their vocal behavior.

L. 378-379: Would it also be possible that visual display is unfavored during new moon, and thus engaging in acoustic advertising would be a strategy of communication? Check if the other references indicate a role of moon phase in the pattern of visual display.

This is a great point. We did not see anything in the our search related to this, but we did add the following line:

It is also possible that because there is less available moonlight during new moon periods great argus rely less on visual displays and more on vocal communication.
